# Development and Validation of Liquid Chromatography-Tandem Mass Spectrometry Method for Pharmacokinetic Evaluation of 7β-(3-Ethyl-cis-crotonoyloxy)-1α-(2-methylbutyryloxy)-3,14-dehydro-Z-notonipetranon in Rats

**DOI:** 10.3390/molecules25081774

**Published:** 2020-04-13

**Authors:** Nae-Won Kang, Jae-Young Lee, Kwangho Song, Min-Hwan Kim, Soyeon Yoon, Duy-Thuc Nguyen, Sungho Kim, Yeong Shik Kim, Dae-Duk Kim

**Affiliations:** 1 College of Pharmacy and Research Institute of Pharmaceutical Sciences, Seoul National University, Seoul 08826, Korea; nwkangkr@snu.ac.kr (N.-W.K.); mhkim305@naver.com (M.-H.K.); sokyu123@naver.com (S.Y.); nguyenduythuc92@snu.ac.kr (D.-T.N.); sungho122333@naver.com (S.K.); 2 College of Pharmacy, Chungnam National University, Daejeon 34134, Korea; jaeyoung@cnu.ac.kr; 3 Natural Products Research Institute, College of Pharmacy, Seoul National University, Seoul 08826, Korea; siwcazb@snu.ac.kr (K.S.); kims@snu.ac.kr (Y.S.K.)

**Keywords:** ECN, *Tussilago farfara* Linnaeus, 7*β*-(3-Ethyl-cis-crotonoyloxy)-1*α*-(2-methylbutyryloxy)-3,14-dehydro-*Z*-notonipetranone, LC-MS/MS, validation, pharmacokinetics

## Abstract

Recently, potent neuroprotective and anti-diabetic effects of 7*β*-(3-Ethyl-cis-crotonoyloxy)-1*α*-(2-methylbutyryloxy)-3,14-dehydro-*Z*-notonipetranone (ECN), a sesquiterpenoid isolated from *Tussilago farfara* Linnaeus, have been elucidated. To facilitate further pre-clinical evaluation in rats, an analytical method for the determination of ECN in rat plasma was developed and optimized by using liquid chromatography-tandem mass spectrometry (LC-MS/MS). Plasma samples were pretreated by the protein precipitation method with an acetonitrile solution of losartan (LST) as the internal standard. Chromatographic separation was performed using a an Octadecyl-silica (ODS) column (2.6 µm, 100 x 4.6 mm) in the isocratic mode. The mobile phase, comprising 10 mM ammonium formate in water pH 5.75) and acetonitrile (11:89, *v*/*v*), was eluted at a flow rate of 0.4 mL/min. Mass spectrometric detection was performed in the multiple reaction monitoring mode with positive electrospray ionization, and the mass transitions of ECN and LST were *m/z* 431.3 to 97.3 and *m/z* 423.1 to 207.2, respectively. The calibration curves of spiked plasma samples were linear in the 10.0–10,000 ng/mL range (*r*^2^ > 0.996). The lower limit of quantification (LLOQ) was determined as 10.0 ng/mL. Validation was conducted in the LLOQ, and three quality control (QC) sample levels (10.0, 25.0, 3750, and 7500 ng/mL) were studied. Among them, the relative standard deviation for the within- and between-run precisions was under 9.90%, and the relative error of the accuracies was within the −8.13% to 0.42% range. The validated method was successfully employed to investigate the pharmacokinetic properties of ECN in rats, which revealed the linear pharmacokinetic behavior of ECN for the first time.

## 1. Introduction

*Tussilago farfara* Linnaeus (Asteraceae) is a perennial herbaceous plant, which is widespread across East Asia, North Africa, Siberia, and Europe, and contains various active compounds that can alleviate several disease-related symptoms [1,2,3]. Particularly, the sesquiterpenoids, terpenoids, steroids, and flavonoids isolated from *Tussilago* are well known for their antioxidant [4], anti-inflammatory [5,6,7], anti-microbial [8], anti-tuberculosis [9], anti-tussive [10,11], anti-α-glucosidase [12], anti-platelet [13], anti-cancer [14], and neuroprotection effects [15]. In addition, some terpenoids isolated from this plant displayed cellular protection activities against various oxidative stresses and xenobiotic damages [16,17].

Among these compounds, the sesquiterpenoid 7*β*-(3-ethyl-cis-crotonoyloxy)-1*α*-(2-methylbutyryloxy)-3,14-dehydro-*Z*-notonipetranone (ECN) displayed cytoprotection effects in microglial cells, by inhibition of nitric oxide, prostaglandin E2, and tumor necrosis factor-α productions via nuclear factor- κB (NF-κB) pathway suppression [18]. Importantly, its nuclear factor erythroid 2–related factor 2 (Nrf2)-mediated neuroprotective activity in mice was demonstrated by our group [19]. Furthermore, ECN displayed inhibition of diacylglycerol acyltransferase (DGAT1) activity, which suggests its potential therapeutic application for the treatment of obesity and type II diabetes [20].

Owing to its potent pharmacological activities, a method for the extraction and isolation of ECN from *T. farfara* has been investigated [21]. However, pre-clinical studies investigating ECN’s pharmacokinetic properties have not been reported yet, and suitable analytical methods for its determination in biological fluids are not available. To address these limitations, we report herein the development and validation of a quantitative analytical method using liquid chromatography-tandem mass spectrometry (LC-MS/MS) for the determination of ECN in rat plasma samples. The pharmacokinetics of ECN after intravenous and oral administration in rats were studied by employing the developed method.

## 2. Results and Discussion

### 2.1. Optimization of LC-MS/MS Conditions

**Figure 1:**
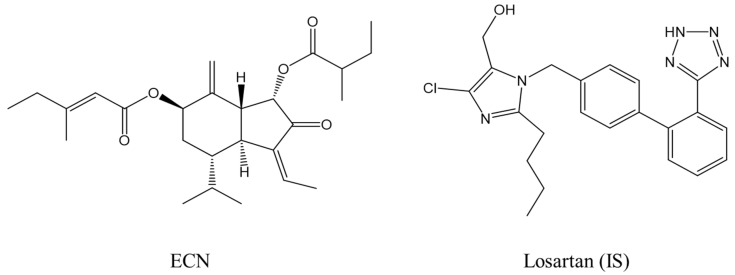
Chemical structures of ECN and losartan (IS).

**Figure 2:**
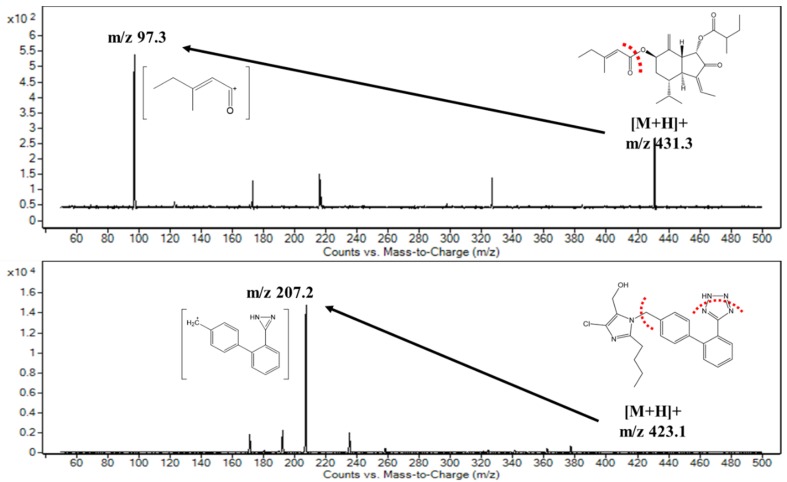
Proposed fragmentation patterns and tandem mass spectra of ECN and losartan (IS).

We began with the optimization of the signal intensities for analyzing ECN and losartan (LST) under MS conditions (Figure 1; molecular weight: 430.3 and 422.1 g/mol, respectively). We used losartan as an internal standard (IS) because of its high stability in plasma [22]. The results indicated that both analytes exhibited higher intensity during mass scanning in the ESI positive mode than in the negative mode, where protonated molecular ions were dominantly observed with an *m/z* of 431.3 for ECN and an *m/z* of 423.1 for LST. Therefore, these [M+H]^+^ ions were selected as the precursor ions, which were then fragmented using the product ion scan mode to single out daughter ions for further analysis in the MRM mode. As shown in Figure 2, the highest intensity of the product ion signals was observed at *m/z* 97.3 for ECN and *m/z* 207.2 for LST. Thus, the mass transitions from the protonated ions to these product ions (i.e., *m/z* 431.3 → 97.3 for ECN and *m/z* 423.1 → 207.2 for LST) were selected for further optimization in MRM mode. Notably, the product ion signal at *m/z* 97.3 was also observed in the product ion spectrum of tussilagone [23], which suggests that this ion may have been derived from the cleavage of the ester bond and the subsequent release of the 3-methylpenten-2-one ion (Figure 2). The fragmentation pattern of LST was similar to that reported in a previous study [24]. Next, we optimized the acquisition conditions to improve the signal intensity. The ionization source settings were optimized by varying the parameters manually, and the values for the nebulizer pressure, gas temperature, and capillary voltage were set at 30.0 psi, 350 °C, and 4.00 kV, respectively, and these conditions afforded the highest signal intensity among the tested conditions (Table S1).

Having optimized the MS conditions, we turned our attention to the optimization of the LC conditions, which were optimized for the peak area, shape, and retention time. Various types of LC columns, including Kinetex C8 5 μ 100 Å (250 × 4.6 mm; Phenomenex), Poroshell 120 EC-C18 2.7 μ (50 × 4.6 mm; Agilent), Chirex S-VAL and DNAn (50 × 4.6 mm; Phenomenex), Synergi Max-RP 4 μ 80 Å (75 × 4.6 mm; Phenomenex), and Synergi Hydro-RP 4 μ 80 Å (75 × 2.0 mm; Phenomenex), were tested (data not shown). Among them, Kinetex C18 2.6 μ 100 Å column (100 × 4.6 mm; Phenomenex) was selected for further investigation, as it provided the highest peak intensity and resolution with a symmetric peak shape, compared with that of the others. The mobile phase composition (10 mM ammonium formate in water vs. acetonitrile (ACN)) was also optimized by varying the ratio of ACN from 85% to 95%. Lower than 85% of ACN resulted in a long retention with a low peak area (data not shown), and the pH of water containing 10 mM ammoniuim formate was 5.75. From the optimization results listed in Table S2, the relative peak area of ECN exhibited the maximum value at the composition with 89% of ACN. Thus, this ratio was selected for further analysis based on separation parameters, including the peak resolution, symmetry, theoretical plate number, and peak area.

### 2.2. Method Validation

#### 2.2.1. Selectivity

**Figure 3:**
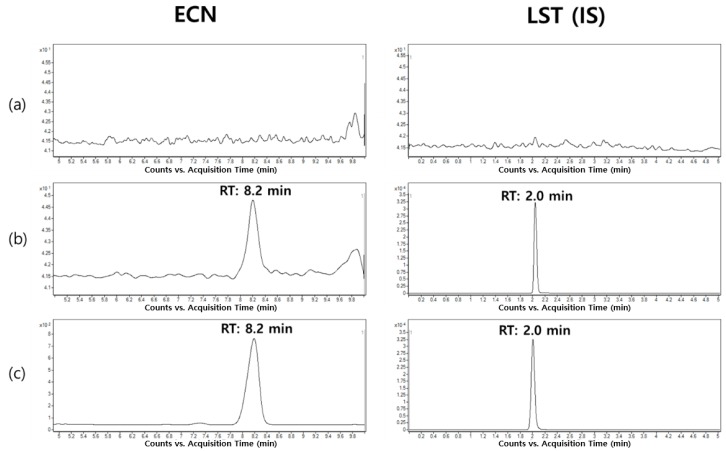
Representative chromatograms of ECN and losartan (LST) in rat plasma samples. (**a**) blank rat plasma sample, (**b**) LLOQ samples (10.0 ng/mL), and (**c**) rat plasma sample collected at 5 min after intravenous administration (dose: 3 mg/kg).

The representative MRM chromatograms of ECN and LST are shown in Figure 3. Compared with the plasma samples at the LLOQ concentration, the blank rat plasma exhibited no significant interference for both analytes within the analytical windows. In addition, the signal-to-noise (S/N) ratio values for ECN at the LLOQ concentration were 11.9 ± 1.6 (*n* = 4; mean ± standard deviation). Furthermore, consistent retention times of ECN (8.2 min) and LST (2.0 min) were observed for both ECN-spiked samples and the in vivo experimental samples obtained after intravenous injection of ECN, which confirmed the selectivity of the developed analytical method.

#### 2.2.2. Linearity

The calibration curves for ECN were constructed with nine different concentration levels (10.0–10,000 ng/mL) using 1/*x*^2^-weighted least squares regression, and are represented by the following equation: *y* = *(0.0000326 ± 0.00000122) x* + *(0.000199 ± 0.000156)* (*n* = 3; mean ± standard deviation). Notably, the correlation coefficients (*r*^2^) of the calibration curves were higher than 0.996, which indicated good linearity of the developed method for ECN analysis.

#### 2.2.3. Within- and Between-Run Precision and Accuracy

Having demonstrated good selectivity and linearity for ECN measurement, we assessed the precision and accuracy of the developed method by evaluating the RSD and RE values, respectively (Table 1). The within-run RSD and RE values of the tested samples (LLOQ and QC samples) ranged from 2.59% to 9.90% and −7.58% to 0.42%, respectively, which were within the acceptable ranges set forth in the U.S. Food and drug administration (FDA) guideline [25]. In the between-run test, the RSD and RE values of the LLOQ and QC samples ranged from 3.74% to 7.57% and −8.13% to −5.56%, respectively. Overall, these results demonstrate good precision and accuracy of the developed method.

#### 2.2.4. Matrix Effect and Extraction Recovery

The results from the matrix effect and extraction recovery studies of ECN and LST are listed in Table 2. The proteins from the plasma samples were removed using a solvent precipitation method, which is relatively straightforward and rapid compared to the other methods [26]. Although this pretreatment method retains solvent-soluble compounds, such as lipids and fatty acids, in the analytical sample [27], negligible matrix effects were observed on the ECN analysis (ranging from 83.3 ± 7.7% to 93.1 ± 0.9%) in this study. Moreover, moderate extraction recovery values ranging from 58.5 ± 3.9% to 77.7 ± 5.0% were achieved with this pretreatment method. On the other hand, the matrix effect and extraction recovery of LST at its working concentration (80.0 ng/mL) were 70.3 ± 1.1% and 99.5 ± 0.9%, respectively. These results demonstrate excellent extraction efficiencies and minimal matrix interferences in the developed method.

#### 2.2.5. Pre- and Post-Preparative Stability

The stability of ECN in the plasma samples was tested by exposing them to various conditions, both before and after the pretreatment process (Table 3). To evaluate the pre-preparative stability, three different conditions were simulated, which were bench-top exposure at room temperature, freeze-thaw cycles, and long-term storage in a freezer. In our preliminary study, the plasma samples exposed at room temperature for 90 min exhibited relatively low stability, for which the RSD and RE values ranged from 0.30% to 15.6% and −28.9% to −25.0%, respectively (Table S3). These low stabilities can be attributed, in part, to the presence of the ester bond in the ECN structure. As ester bonds have a high propensity for hydrolysis by the esterases in the plasma [28,29], a low-temperature condition (4 °C) that can inhibit the enzyme activity was maintained during the solvent precipitation process. As shown in Table 3, the samples incubated at 4 °C for 90 min displayed acceptable RSD and RE values, ranging from 1.95% to 10.3% and −5.70% to 1.13%, respectively. The freeze-thaw stability study results exhibited RE values from −9.50% to −6.47% with acceptable RSD values. However, the RE values for long-term stability ranged from −34.8% to −26.5%, which indicated that the long-term storage of the plasma sample is not suitable. Thus, the plasma samples obtained from the pharmacokinetic studies were pretreated with cold ACN immediately after collection from rats according to the method described in Section 3.5. To assess the post-preparative stability, the analytical samples were maintained in the autosampler (10 °C) for 24 h. The RE values of the autosampler-kept LLOQ and QC samples ranged from −12.9% to 4.88% with RSD values under 10.5%, which indicated that the analytical samples were stable under these conditions. Overall, conditions for the safe preparation and manipulation of the samples for reliable analysis of ECN were defined in this study.

### 2.3. Pharmacokinetic Studies

**Figure 4:**
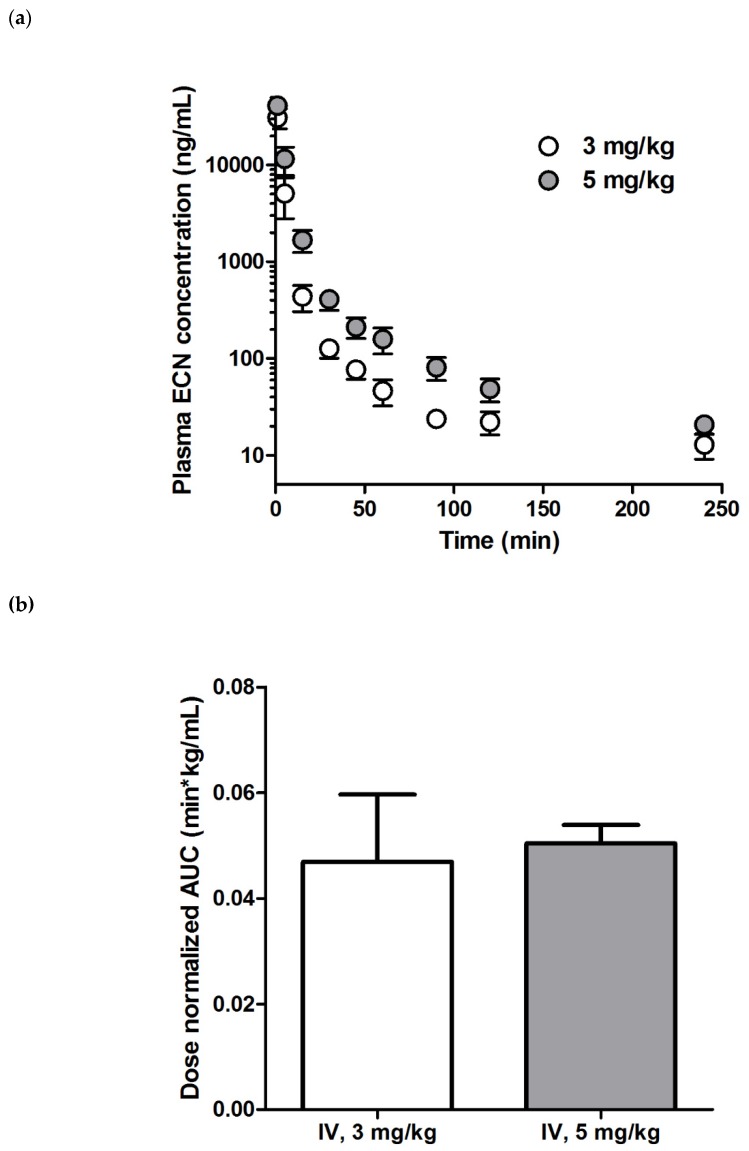
The ECN pharmacokinetics in rats was studied. Plasma concentration–time profiles of ECN after intravenous (IV) administration of a 3 or 5 mg/kg dose (**a**) and the dose-normalized AUC_last_ value of each group (**b**) are presented. Each point indicates mean ± SD (*n* = 3).

With the method validation complete and the various conditions for the robust analysis of ECN defined, we investigated the pharmacokinetic properties of ECN in rats using the established analytical method. The plasma samples with concentrations higher than ULOQ were diluted and re-analyzed using the validated method described in Section 3.7, for which the RE and RSD values were in the acceptable ranges (Tables S4). The profiles for the ECN plasma concentration vs. time after a single ECN IV administration are shown in Figure 4a and the corresponding pharmacokinetic parameters are listed in Table 4. The concentration of ECN exhibited a multi-exponential decline in the plasma after bolus injection. Notably, the t_1/2_, CL_p_, and V_d,ss_ values of both groups (i.e., 3 or 5 mg/kg administration) were not significantly different. Moreover, the dose-normalized AUC_last_ of both groups also exhibited almost similar values (0.0498 ± 0.0128 and 0.0506 ± 0.0035 min·kg/mL for 3 and 5 mg/kg, respectively), indicating that the exposure of ECN increased proportionally with the dose (Figure 4b). These results suggested that the linear kinetics of ECN were within the tested dose range. Previously, Liu et al. reported the pharmacokinetic parameters of tussilagone, which is another major compound isolated from *Tussilago farfara* L. that has a structure similar to that of ECN, after IV administration at a dose of 5 mg/kg [30]. Interestingly, the mean CL_p_ value for ECN (0.021 ± 0.0063 L/min/kg) at the same dose was much lower than that of tussilagone (0.04 ± 0.01 L/min/kg), thereby resulting in a 1.9-fold higher mean AUC value of ECN.

Next, we studied the oral (PO) administration of ECN in rats at the same dose (5 mg/kg dissolved in the same vehicle used for IV administration). However, no significant ECN signals higher than the LLOQ were observed in this study (data not shown). It has been reported that tussilagone also showed very low absolute bioavailability when administered orally (1.31%; 200 mg/kg PO vs. 5 mg/kg IV) due to its poor solubility and metabolism in the liver. Considering the similarity of the molecular structures of ECN and tussilagone, the low oral bioavailability of ECN could be attributed to similar causes [29], and further studies are required to fully elucidate the exact reasons for this phenomenon.

## 3. Materials and Methods

### 3.1. Materials

ECN (purity ≥ 97.0%) was isolated according to our previous report [21]. Losartan (LST) potassium (IS; purity ≥ 99.5%) and ammonium formate (purity ≥ 99.0%) were purchased from Sigma-Aldrich (St. Louis, MO, USA). Acetonitrile (ACN; HPLC grade), ethanol (EtOH; purity: 99.5%), and water (HPLC grade) were obtained from Thermo Fisher Scientific (Waltham, MA, USA). Normal saline (NS) was purchased from Daihan Pharm. Co., Ltd. (Seoul, Korea). Dimethyl sulfoxide (DMSO) was obtained from Daejung Chemicals & Metals Co., Ltd. (Siheung, Korea). All other reagents were of analytical grade and were acquired from commercial sources.

### 3.2. Animals Studies

Male Sprague–Dawley (SD) rats (body weight: 300 ± 10 g) were obtained from Orient Bio (Seongnam, Korea). The rats were reared in a light-controlled room at 22 ± 2 °C and relative humidity of 55 ± 5% in the Animal Center for Pharmaceutical Research, College of Pharmacy, Seoul National University (Seoul, Korea). The pharmacokinetic study protocols (SNU-190527-3) were approved by the Animal Care and Use Committee of the Seoul National University (Seoul, Korea).

### 3.3. Apparatus and Conditions

Chromatographic separation of ECN was performed using an HPLC system (Agilent Technologies 1260 Infinity; Agilent Technologies, Palo Alto, CA, USA) equipped with a G1367E autosampler, a G1312B binary pump, a G1316C thermostated column compartment, and a G1330B thermostat. An aliquot (5 µL) of the analytical sample was injected into a Kinetex C18 2.6 μ 100 Å column (100 × 4.6 mm; Phenomenex, CA, USA) with a C18 guard column (4 × 2.0 mm; Phenomenex) at 25 °C. Isocratic elution was performed at a flow rate of 0.4 mL/min with a total run time of 10 min. The mobile phase was composed of 10 mM ammonium formate in water (pH 5.75) and ACN (11:89, *v*/*v*).

The mass spectrometric detection was performed using an Agilent Technologies 6430 Triple Quad LC/MS system in the positive electrospray ionization (ESI) mode. The optimized ESI source parameters were manually set to the following values: Gas temperature, 350 °C; gas flow, 11 L/min; nebulizer pressure (nitrogen), 30 psi; and capillary voltage, 4.00 kV. The multiple reaction monitoring (MRM) mode was used at the unit resolution for both Q1 and Q3 mass filters. The optimized molecular mass transitions of the precursor to product ion/fragmentor voltages (V)/collision energies (eV) for ECN and LST were *m/z* 431.3 → 97.3/105/13 and *m/z* 423.1 → 207.2/115/20, respectively. The data were acquired and processed using Mass Hunter Workstation Software (Version B.05.00; Agilent Technologies).

### 3.4. Preparation of Calibration Curve and Quality Control Samples

The stock solutions of ECN (15.0 mg/mL in DMSO) and LST (1.00 mg/mL in the co-solvent: ACN and DMSO (1:1, *v*/*v*)) were prepared by vortex mixing and subsequent bath sonication. The working solutions of ECN were prepared by serial dilution of the stock solutions with ACN to be the 20-fold concentration of the calibration standard and quality control (QC) samples. The LST working solutions were prepared with ACN at a concentration of 2.00 µg/mL (20-fold concentrated). The calibration standard and the quality control samples were prepared by adding the working solution (5 µL; 4 °C) to the blank rat plasma (95 µL; 4°C) to make up the final concentrations of the calibration standard samples to 10000, 2000, 1000, 500, 200, 100, 50.0, 20.0, and 10.0 ng/mL; and QC samples to 7500, 3750, and 25.0 ng/mL. The concentration of the low-QC sample was selected based on the FDA guideline, where a value lower than three-fold of the LLOQ is recommended. The high-QC concentration was set to be 0.75-fold of the highest concentration in the calibration curve. The middle-QC concentration was determined to be half of the high-QC concentration.

### 3.5. Sample Pretreatment

All plasma samples (i.e., blank plasma, calibration standard, and QC samples) were manipulated at 4 °C to prevent degradation. An aliquot (50 µL) of the plasma sample was vortex-mixed with ACN (200 µL) containing LST (as IS, 100 ng/mL) for 5 min, and the mixture was centrifuged at 16,000 *g* for 5 min. The supernatant (150 µL) of each sample was transferred into a sample vial and was placed in the autosampler of the LC-MS/MS system mentioned in Section 3.3.

### 3.6. Method Validation

All validation procedures for ECN analysis in rat plasma were carried out in accordance with the US Food and Drug Administration (FDA) guidelines [25]. The rat plasma samples were obtained from the heparinized (20 U/mL) rat blood samples.

#### 3.6.1. Selectivity

The selectivity of the developed analytical method was evaluated by comparing the chromatograms of blank plasma samples from six different rats. The presence of interfering signals during the ECN and LST acquisitions was investigated.

#### 3.6.2. Linearity and LLOQ

The linearity of the calibration curve was assessed over the 10.0–10000 ng/mL concentration range and the curve (*y* = a*x* + b) was constructed by plotting the peak area ratios of ECN with the IS (*y*) versus the corresponding nominal concentrations of calibration standards (*x*) using least-squares regression with a weighting factor of 1/*x*^2^. The LLOQ was determined as the lowest concentration that can be quantified with acceptable accuracy, with a relative error (RE) within ±20%, and a relative standard deviation (RSD) of precision under 20%.

#### 3.6.3. Within- and Between-Run Precision and Accuracy

Six replicates of the LLOQ (10.0 ng/mL) and QC samples (low-QC: 25.0 ng/mL; middle-QC: 3750 ng/mL; and high-QC: 7500 ng/mL) were analyzed in three different runs to evaluate the within- and between-run precision and accuracy. Precision was assessed in terms of RSD, with an acceptable range of under 20% for the LLOQ samples and 15% for the QC samples. Accuracy was evaluated based on RE, for which the acceptable range was within ±20% for the LLOQ samples and ±15% for the QC samples.

#### 3.6.4. Matrix Effect and Extraction Recovery

Four replicates of the LLOQ and QC samples were analyzed to assess the matrix effect and extraction recovery of ECN and LST. Each replicate was made from the different blank plasma lots. The matrix effect was determined by comparing the peak areas of the analytes spiked after the extraction with those in the neat solution. The extraction recovery was calculated by comparing the peak areas of analytes spiked before extraction with those spiked after the extraction.

#### 3.6.5. Pre- and Post-Preparative Stability

The stability of ECN was evaluated under five different conditions that the analytes can be exposed to during the analysis procedure. To assess the pre-preparative stability, four replicates of the LLOQ and QC samples were prepared. One set of these samples was maintained at room temperature (bench-top stability) for 90 min, and another at 4 °C (representing our sample preparation condition) for the same time. The third set was stored at −20 °C for 35 days (long-term stability), and the final set was subjected to three freeze (−20 °C) and thaw (4 °C) cycles (freeze-thaw stability). The post-preparative stability of the samples was also tested by maintaining the analytical samples in the autosampler at 10 °C for 24 h. The RE and RSD values within ±15% and under 15%, respectively, were considered stable.

### 3.7. Sample Dilution

Plasma samples with a concentration above the upper limit of quantification (ULOQ; 10,000 ng/mL) were diluted with the same matrix (blank rat plasma) and were re-analyzed. To validate the dilution method, QC samples with ECN concentrations of 20,000 and 50,000 ng/mL were prepared using the method described in Section 3.4. The samples were diluted 10-fold with the blank rat plasma and were pretreated according to the method described in Section 3.5. The precision and accuracy were assessed in terms of RSD (acceptable range: under 15%) and RE (acceptable range: within ±15%), respectively.

### 3.8. Pharmacokinetic Study

The pharmacokinetic properties of ECN were investigated in triplicate, in SD rats. The left femoral artery and vein were cannulated into with polyethylene tubes (Intramedic™ PE-50; Becton Dickinson Diagnostics, NJ, USA) under Zoletil (Virbac, Carros, France) anesthesia (50 mg/kg, intramuscular injection). A solution of ECN in a DMSO/EtOH/NS mixture (1:1:1, *v*/*v*/*v*) was intravenously (IV) injected through the femoral vein over 30 s (3 or 5 mg/kg) or was orally administered (5 mg/kg), after which blood samples (150 μL) were collected from the femoral artery at predetermined times (0 as a blank, 1, 5, 15, 30, 45, 60, 90, 120, and 240 min), and an equivalent volume of normal saline containing heparin (20 U/mL) was injected intravenously at each time point. The collected blood samples were centrifuged at 16,000 *g* for 2 min (4 °C), and an aliquot (50 μL) of the supernatant was collected and pretreated immediately according to the method described in Section 3.5. The ECN concentration was determined using the analytical method described in Section 3.3. Samples with a concentration above the ULOQ were diluted and re-analyzed according to the method described in Section 3.7. Pharmacokinetic parameters calculated by non-compartmental analysis using the WinNonlin software (Build 8.1.0.3530; Phoenix) were as follows: The area under the plasma concentration–time curve from zero to time last (AUC_last_), area under the plasma concentration–time curve from zero to time infinity (AUC_inf_), terminal half-life (t_1/2_), total body plasma clearance (CL_p_), and apparent volume of distribution at steady state (V_d,ss_). A comparison of the dose-normalized AUC_last_ values among the IV groups was conducted using Student’s *t*-test, where *p* < 0.05 was considered significantly different.

## 4. Conclusions

An LC-MS/MS method for the determination of ECN in rat plasma was successfully developed and validated in accordance with the US FDA guidelines. The validation parameters included the selectivity, linearity, accuracy, precision, and stability. The validated method was then applied to investigate the ECN pharmacokinetics in rats, which revealed the linear pharmacokinetic behavior of ECN for the first time. In addition, ECN exhibited higher systemic exposure than tussilagone at the same dose, which highlights its potential as a promising drug candidate. Overall, the analytical method established in this study will facilitate further preclinical studies of ECN.

## Figures and Tables

**Table 1:** Within- and between-run precision and accuracy for ECN measurement in rat plasma.

Nominal Concentration (ng/mL)	Within-Run (*n* = 6)			Between-Run (*n* = 3)		
	Concentration Determined (ng/mL)	RSD^a^ (%)	RE^b^ (%)	Concentration Determined (ng/mL)	RSD (%)	RE (%)
10.0	9.24	9.90	−7.58	9.27	5.97	−7.33
25.0	24.3	5.12	−2.89	23.0	7.57	−8.13
3750	3766	3.11	0.42	3542	5.61	−5.56
7500	7215	2.59	−3.80	6977	3.74	−6.98

^a^ RSD (%) = (standard deviation of determined concentrations/mean concentration) × 100. ^b^ RE (%) = ((determined concentration—nominal concentration)/nominal concentration) × 100.

**Table 2:** Matrix effect and extraction recovery of ECN.

Nominal Concentration (ng/mL)	Matrix Effect (%) ^a^	Extraction Recovery (%) ^b^
**ECN**		
10.0	83.3 ± 7.65	62.6 ± 4.56
25.0	86.7 ± 4.28	58.5 ± 3.88
3750	89.6 ± 1.50	73.4 ± 1.41
7500	93.1 ± 0.86	77.7 ± 5.03
**Losartan (IS)**		
80.0	70.3 ± 1.10	99.5 ± 0.93

^a^ Matrix effect (%) = ((peak area of analyte spiked after extraction)/(peak area of analyte in neat solution)) × 100. ^b^ Extraction recovery (%) = ((peak area of analyte spiked before extraction)/(peak area of analyte spiked after extraction)) × 100. Data are presented as mean ± SD (*n* = 4).

**Table 3:** Pre- and post-preparative stability of ECN.

Nominal Concentration (ng/mL)	Pre-preparative Stability (*n* = 4)									Post-preparative Stability (*n* = 4)		
	4°C Stability ^a^			Long-term Stability ^b^			Freeze-thaw Stability ^c^			Autosampler Stability^d^		
	Mean	RSD (%)	RE (%)	Mean	RSD (%)	RE (%)	Mean	RSD (%)	RE (%)	Mean	RSD (%)	RE (%)
10.0	9.89	10.3	−1.13	7.35	20.4	−26.5	9.05	18.0	−9.50	10.5	3.94	4.88
25.0	24.7	4.06	−1.25	16.3	8.43	−34.8	23.0	18.1	−7.90	23.8	10.5	−4.87
3750	3575	1.95	−4.67	2581	3.65	−31.2	3508	1.36	−6.47	3351	2.04	−10.6
7500	7073	3.11	−5.70	5502	2.77	−26.6	6953	1.50	−7.30	6537	0.97	−12.9

^a^ After maintaining at 4 °C for 90 min; ^b^ After storing at −20 °C for 35 days; ^c^ After three freeze (−20 °C) and thaw (4 °C) cycles; ^d^ After maintaining at 10 °C for 24 h in the autosampler.

**Table 4:** Pharmacokinetic parameters of ECN after intravenous (IV) administration in rats.

Parameter	IV (3 mg/kg)	IV (5 mg/kg)
AUC_last_ (μg∙min/mL)	149 ± 42	253 ± 21
AUC_inf_ (μg∙min/mL)	152 ± 42	256 ± 21
t_1/2_ (min)	80.0 ± 6.9	84.4 ± 15.5
CL_p_ (mL/min/kg)	21.0 ± 6.3	19.7 ± 1.6
V_d,ss_ (mL/kg)	203 ± 25	252 ± 51

Data are presented as mean ± SD (*n* = 3).

## Supplementary Materials

The following are available online. Table S1: Optimization of ionization source settings, Table S2: Optimization of mobile phase composition, Table S3: ECN stability at room temperature over 90 min, Table S4: Precision and accuracy of sample dilution method.
