# Supplementary material for: Development and Validation of Liquid Chromatography-Tandem Mass Spectrometry Method for Pharmacokinetic Evaluation of 7β-(3-Ethyl-cis-crotonoyloxy)-1α-(2-methylbutyryloxy)-3,14-dehydro-Z-notonipetranon in Rats
Source: Molecules. 2020 Apr 13;25(8):1774. doi: 10.3390/molecules25081774 (PMC7221868; doi:10.3390/molecules25081774)
Supplement: Supplementary file 1 [file molecules-25-01774-s001.pdf]

Supplementary Information

Development and Validation of Liquid Chromatography-Tandem Mass Spectrometry Method for Pharmacokinetic Evaluation of 7β-(3-Ethyl-cis-crotonoyloxy)-1α-(2-methylbutyryloxy)-3,14-dehydro-Z-notonipetranon in Rats

Nae-Won Kang [1,†], Jae-Young Lee [2,†], Kwangho Song [3], Min-Hwan Kim [1], Soyeon Yoon [1] and Duy-Thuc Nguyen [1], Sungho Kim [1], Yeong Shik Kim [3] and Dae-Duk Kim [1,*]

[1] College of Pharmacy and Research Institute of Pharmaceutical Sciences, Seoul National University, Seoul 08826, Republic of Korea

[2] College of Pharmacy, Chungnam National University, Daejeon 34134, Republic of Korea

[3] Natural Products Research Institute, College of Pharmacy, Seoul National University, Seoul 08826, South Korea

* Correspondence: ddkim@snu.ac.kr; Tel.: +82 2 880 7870; fax: +82 2 873 9177

† These authors have equally contributed.

Table 1. Optimization of ionization source settings.

Condition No.	Nebulizer pressure (psi)	Gas temperature (℃)	Capillary voltage (kV)	Change in peak area by fold[a]
1	15	300	4	1.00
2	15	310	4	1.06
3	15	320	4	1.13
4	15	330	4	1.24
5	15	340	4	1.21
6	15	350	4	1.29
**7**	**30**	**350**	**4**	**1.67**
8	50	350	4	1.64
9	60	350	4	1.63
10	30	350	5	1.59
11	30	350	6	1.58

[a] Compared to condition No. 1.

Table 2. Optimization of mobile phase composition.

Condition No.	ACN (%)	Change in peak area by fold[a]
1	15	1.00
2	14	1.03
3	13	1.06
4	12	1.18
**5**	**11**	**1.23**
6	10	1.20
7	9	1.19
8	8	1.19
9	7	1.14
10	6	1.06
11	5	0.97

[a] Compared to condition No. 1.

Table 3. ECN stability at room temperature over 90 min.

Nominal concentration (ng/mL)	Mean	RSD (%)	RE (%)
10.0	7.11	15.6	-28.9
25.0	18.8	2.90	-25.0
3750	2776	1.52	-26.0
7500	5588	0.30	-25.5

Table 4. Precision and accuracy of sample dilution method.

Nominal concentration (ng/mL)	Mean	RSD (%)	RE (%)
20000	21300	3.26	6.52
50000	56870	0.885	13.7
